# An atypical reproductive cycle in a common viviparous Asia Agamid *Phrynocephalus vlangalii*

**DOI:** 10.1002/ece3.1783

**Published:** 2015-10-19

**Authors:** Yayong Wu, Jinzhong Fu, Bisong Yue, Yin Qi

**Affiliations:** ^1^ Herpetological DepartmentChengdu Institute of BiologyChinese Academy of SciencesChengduSichuan610041China; ^2^ College of Life ScienceSichuan UniversityChengdu610064China; ^3^ University of Chinese Academy of SciencesBeijing100049China; ^4^ Departments of Integrative BiologyUniversity of GuelphGuelphOntarioN1G 2W1Canada

**Keywords:** Asynchronous vitellogenesis and gestation, *Phrynocephalus vlangalii*, relative clutch mass, reproductive cycle, sexual maturity

## Abstract

Viviparous lizards living in cold climate of high altitude often exhibit atypical reproductive cycles, in which mating and fertilization occur synchronously and annually with parturition occurring at the end of the year. Nevertheless, detailed case studies on atypical reproductive cycles are few. Using anatomical data combined with behavioral observations, we examined the reproductive cycle of a common Asian agamid, *Phrynocephalus vlangalii*, from a high‐elevation area in Sichuan, China. Male spermiation of *P. vlangalii* occurred in May, and spermatogenesis began in June and reached a maximum in October. For females, ovulation and fertilization occurred in May, and females developed gestation and pregnancy in 3 months from June to August, without vitellogenesis during this period. Females gave birth synchronously in late August, then vitellogenesis began and lasted until May of the next year. All adult males and females were synchronized in the same reproductive condition each month. The synchronous and annual reproductive cycle of *P. vlangalii* clearly represents an atypical cycle. The male courtship and mating behaviors were concordant with gonadal cycle and mainly happened in May and June. Despite the short growth period for neonates, they had a high over‐winter survival rate of 84.4%, suggesting that autumn parturition did not generate high costs to this reproductive cycle. We propose that the high over‐winter survival rate of neonates is likely linked with female delayed sexual maturity, female asynchronous vitellogenesis and gestation, large relative clutch mass (RCM), and adult‐offspring burrow sharing behavior during hibernation.

## Introduction

The timing of events during a reproductive cycle affects multiple life history traits that are acted on by natural selection to maximize reproduction of offspring (Roff 2002; Shine 2005). For viviparous lizards, the reproductive cycle is largely dependent on abiotic factors, such as precipitation, temperature and photoperiod (Shine 2005; Ramírez‐Pinilla et al. 2009; Clusella‐Trullas et al. 2011). For example, precipitation and temperature in tropical zones are consistently high (Clusella‐Trullas et al. 2011), and males tend to reproduce throughout the year, whereas females exhibit a seasonal pattern (Vial and Stewart 1985; Hernández‐Gallegos et al. 2002). In montane areas of central Mexico, asynchronous seasonal reproductive cycle is characteristic of multiple viviparous lizards, including Phrynosomatidae (e.g., *Sceloporus grammicus* from Hidalgo, Mexico, Hernández‐Salinas et al. 2010; *S*. *minor* from La Manzana of Hidalgo, Mexico, Ramirez‐Bautista et al. 2014), Scincidae (e.g., *Eumeces copei*, from Eje Neovolcanico, Mexico, Ramirez‐Bautista et al. 1996), and Xantusiidae (e.g., *Lepidophyma sylvaticum*, Ramirez‐Bautista et al. 2008), in which mating occurs several months prior to ovulation, and parturition occurs in favorable seasons with high availability of food and thermal opportunities (Ramirez‐Bautista et al. 1996; Hernández‐Salinas et al. 2010). In some cold environments of high altitudes, however, viviparous lizards are found to exhibit synchronous seasonal reproductive cycle, in which, mating occurs simultaneously with ovulation, and females give birth in autumn when temperature begins to decline (e.g., *Liolaemus lineomaculatus*, Medina and Ibargüengoytía 2010; *Phymaturus zapalensis*, Boretto and Ibarguengoytia 2009; *P. punae*, Boretto et al. 2007; *S*.* grammicus*, Ramírez‐Bautista et al. 2012; *Zootoca vivipara*, Roig et al. 2000). This reproductive cycle is regarded as “atypical,” because neonates would almost immediately face a long time in hibernation without sufficient time to grow and acquire sufficient energy storage to survive (Medina and Ibargüengoytía 2010; Ramírez‐Bautista et al. 2012). Several hypotheses have been proposed to explain the adaptation of atypical reproductive cycle, such as thermal behavior adjustment, asynchrony in vitellogenesis and gestation, prolonged reproductive cycles, and plasticity in frequency of reproduction (Olsson and Shine 1999; Shine 2005). Nevertheless, direct evidence testing those hypotheses is lacking (Ibargüengoytía 2004; Boretto and Ibarguengoytia 2006; Shine 2006).

Offspring survival is one of the most important indicator of reproductive cycle adaptation, and many viviparous lizards regulate their sexual mature age, gonadal cycle, and energy to improve their offspring survival (Jones et al. 1997; Ibargüengoytía 2004; Shine 2005; Piantoni et al. 2006; Boretto and Ibarguengoytia 2009). Delaying sexual maturity with large female body size has been found in many high‐elevation lizards, and large females tend to give birth to large offspring, thereby increase offspring over‐winter survival (Piantoni et al. 2006; Boretto et al. 2015). The timing of the events of reproductive cycles, like spermatogenesis and vitellogenesis, is also important to offspring survival in viviparous lizards (Guillette and Casas‐Andreu 1980; Ibargüengoytía 2004; Ramírez‐Bautista et al. 2012). Asynchronous gonadal activity, where spermatogenesis and vitellogenesis occur in separate seasons, is common in lizards from cold environments of high altitudes, because of endocrinal and energy constraints (Custodia‐Lora and Callard 2002; Ibargüengoytía 2004; Boretto et al. 2015). Ceasing vitellogenesis during gestation allows more energy to be allocated to embryos (Ibargüengoytía 2004). Furthermore, relative clutch mass (RCM), associated with the amount of energy investment in reproduction, is often adjusted by female lizards to ensure offspring survival (Agrawal et al. 2001; Shine 2005).

Lizards living in cold environments at high elevations provide an excellent system to study atypical reproductive cycle. Many lizards in high elevations are viviparous (Shine 2005), and more importantly, they are under strong environmental stress associated with food availability, thermal opportunities, and offspring survival. These factors likely promote evolution of diverse reproductive cycles (Jin and Liu 2007; Hernández‐Salinas et al. 2010; Medina and Ibargüengoytía 2010; Ramírez‐Bautista et al. 2012). For example, a high proportion of lizards in high‐elevation regions evolve autumn reproductive cycle, in which spermatogenesis and vitellogenesis occur in spring and summer, fertilization and gestation occur in winter, and parturition occurs in spring of the next year (Guillette and Casas‐Andreu 1980; Jiménez‐Cruz et al. 2005). Some lizards in highland adopt spring and summer reproductive cycle, in which spermatogenesis, vitellogenesis, and gestation happen in spring and summer (Gavaud 1986; Pilorge 1987; Powell and Russell 1991). Diverse reproductive cycles might be the result of local adaptation or plastic responses to similar ecological conditions among different species, which may have been the driving forces behind the evolution of atypical reproductive cycle, but the mechanism of this hypothesis is presently unknown (Ibargüengoytía 2004; Ramírez‐Pinilla et al. 2009; Ramírez‐Bautista et al. 2012).

**Figure 1:**
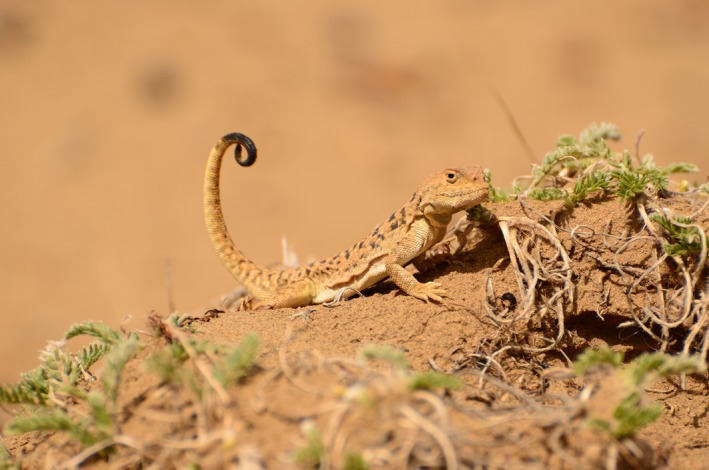
A male *Phrynocephalus vlangalii* curls its tail as dynamic visual signal.

The Qinghai toad‐headed agama (*Phrynocephalus vlangalii*) (Fig. 1) is a common and widespread viviparous lizard at the Qinghai‐Tibetan Plateau. The species occurs along a broad elevation gradient from 2000 to 4500 m a.s.l. (Zhao 1999). Field observation revealed that lizards first emerge from hibernation in early April, and courtship and mating behaviors occur in May and June. Parturition is inferred to occur in August, because of the presence of neonates in late August and September, which is also the period when air temperature begins to decline. Hibernation is inferred to occur in late October, because few lizards are observed after this time. These observations suggest that the reproductive cycle of *P. vlangalii* is likely atypical. In this study, we examined the annual gonadal cycle and age of sexual maturity of *P. vlangalii*. Meanwhile, the adaptive advantages of this reproductive cycle were explored by examining over‐winter survival of their offspring.

## Materials and Methods

### Sampling

**Figure 2:**
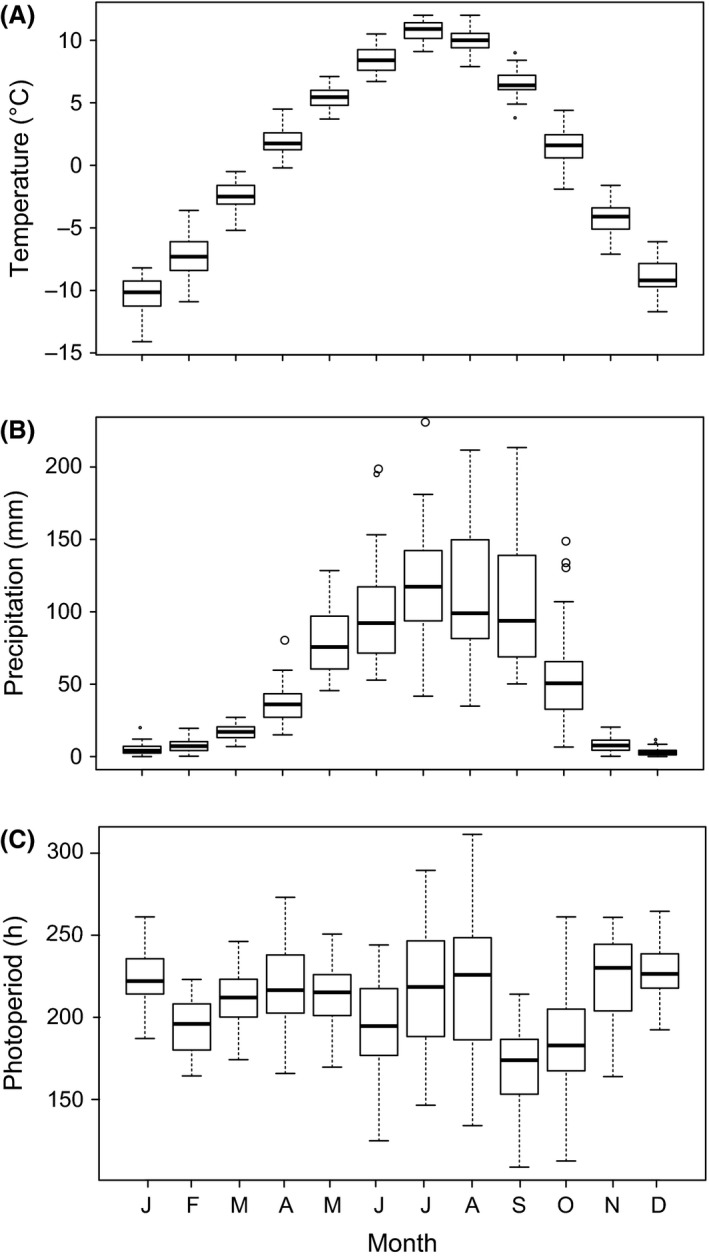
Monthly variation of (A) Temperature, (B) Precipitation, and (C) Photoperiod in the Zoige county, Sichuan, China. Unit of photoperiod “h” represents the number of hours of sunlight, the band inside the box represents the median, the bottom and top of the box represent the first and third quartiles, and the ends of the whiskers represent the minimum and maximum of all of the data. Data were averaged from monthly maximum temperature, precipitation, and photoperiod in Zoige since 1964 to 2004 from China Meteorological Data Sharing Service system (http://cdc.cma.gov.cn/home.do).

Our study site is located near the Xiaman Conservation Station of the Zoige Wetland Nature Reserve in southwestern China (33.71389°N, 102.48543°E; elevation 3475 m a.s.l.). The climate of this area is characterized by a short spring and summer (4 months, from April to July, with high availability of food and thermal opportunities) and long autumn and winter (8 months, with autumn lasting from August to September, and winter lasting from October to March of the next year). The seasonal variation of temperature, precipitation, and photoperiod is presented in Figure 2. The 40‐year period climatic data are collected from China Meteorological Data Sharing Service system (http://cdc.cma.gov.cn/home.do). *Phrynocephalus vlangalii* in Zoige mainly occurs on and around sand dunes with a high density of approximately 3000 lizards/ha. The vegetation is predominantly composed of a shrub (*Salix sclerophylla*) and three grass species (*Kobresia humilis*,* K. prattii,* and *Elymus natans*) (Wu et al. 2002).

To determine male and female reproductive cycle, 4–12 lizards of each sex were captured in 2 days of each month from April to November in 2012. In addition, 15 neonates and 18 subadults (SVL < 50 mm, Wu et al. 2002) were captured in September of 2012 to determine the age of sexual maturity (Table 1). Immediately after capture, lizards were euthanized with an over dose of 5% pentobarbital sodium solution and the snout‐vent length (SVL) was measured to the nearest 0.01 mm using a digital caliper (Mitutoyo, Kanagawa, Japan). Voucher specimens were fixed and preserved with 10% buffered formalin. The adult sex was determined with the presence/absence of hemi‐penis bulge, whereas subadults were determined by examination of the gonads.

**Table 1:** Sample information. Subadults or neonates were collected only in September to ease the size comparison among different age groups, because parturition occurred synchronously in late August and September

Month	Date	Sample size	Male	Female	Subadult	Neonates
April	14‐April	14	10	4	0	0
May	11‐May	22	12	10	0	0
June	16‐June	20	8	12	0	0
July	14‐July	19	10	9	0	0
August	16‐August	16	8	8	0	0
September	15‐September	50	8	9	18	15
October	19‐October	17	8	9	0	0
November	14‐November	18	8	10	0	0

## Morphological examination

Following previous studies, we used three measurements to estimate the gonadal cycle in males, testis volume, diameter of the seminiferous tubules, and stage of spermatogenesis (Ibargüengoytía 2004; Ramírez‐Bautista and Olvera‐Becerril 2004). The maximum and minimum diameter of left testis were measured to estimate testis volume (V) using the formula V = 4/3Πab^2^, where “a” represents 1/2 of maximum testis diameter, and “b” represents 1/2 of minimum testis diameter (Selby 1965). We measured only the left testis because previous studies suggest that testis sizes do not differ between left and right (Ibargüengoytía 2004). Male left testis tissue was dissected and prepared for section using the standard paraffin section technique (Estrada‐Flores et al. 1990; Farias et al. 2006). The diameter of seminiferous tubule was measured under light microscope with an ocular micrometer (Estrada‐Flores et al. 1990; Farias et al. 2006). Six stages of spermatogenesis were defined according to protocols of Radder et al. (2001). We tested for variation in testis volume and diameter of the seminiferous tubules among months using an ANCOVA with SVL as the covariate.

For adult females, follicle size and liver mass were used to track female gonadal cycle (Ramírez‐Bautista et al. 2006). The maximum volume of follicle was measured using the same formula as male testis. Liver mass is an important indicator of reproductive activity, especially for pregnant females because of its link with energy storage (Ramírez‐Bautista and Vitt 1997; Jiménez‐Cruz et al. 2005). The embryo wet mass and yolk dry mass were measured as supplementary information to determine female reproductive cycle, with yolk being dried at 60°C in a drying oven. The variations of follicle size and liver mass across months were tested using ANCOVA, while the SVL was controlled for.

The age of sexual maturity was determined by comparing the body size (SVL) of different age groups. We first determined the sexual maturity by examining the gonads. For males, testis with sperm in seminiferous tubules marks sexual maturity (Goldberg and Lowe 1966; Ramírez‐Bautista et al. 2013). For females, we used the developing embryo or enlarged follicles as indicators of sexual maturity (Ramirez‐Bautista et al. 1998; Ramírez‐Bautista and Olvera‐Becerril 2004). We then classified lizards into three age groups: an adult group consisted of mature males and females, a subadult group consisted of individuals without obvious gonads, and a neonate group consisted of neonates captured in September. The SVL of neonates were used as criterion of zero‐year age. The significance of SVL among different age groups was tested using two‐sample *t*‐test. To validate the age of sexual maturity, twenty neonates were marked in our seminature enclosure since September of 2013, and re‐captured and measured in September of 2014 and 2015, respectively.

### Behavioral observation and offspring fitness

Behavioral observation was carried out in twelve small populations in seminature enclosures (length × width ×height = 5 × 5 × 1.5 m) at the Xiaman Research Station, two kilometers away from our wild site, in 2012. Each population consisted of four males and six females, and the sex ratio and density (4000 lizards/ha) were similar to these of the wild populations. These populations were established primarily for a research project on female intrasexual competition. We recorded the frequency of male courtship and mating behavior using random scan from May to August in 2012 (Martin and Bateson 2007). The values for offspring SVL, offspring mass, litter size, and offspring over‐winter survival were collected based on the twelve enclosures and used as measurement of offspring fitness. In addition, the female prenatal SVL was collected and used as measurement of female size. The female RCM was calculated by the clutch mass/female postpartum mass (Shine 1992) and used to measure the amount of female energy investment in reproduction. The relationship between female size and offspring fitness was tested using Pearson's correlation or Spearman's rank correlation test. All analyses were carried out in R version 3.1.2 (R Development Core Team 2011).

## Results

### Gonadal cycle

**Figure 3:**
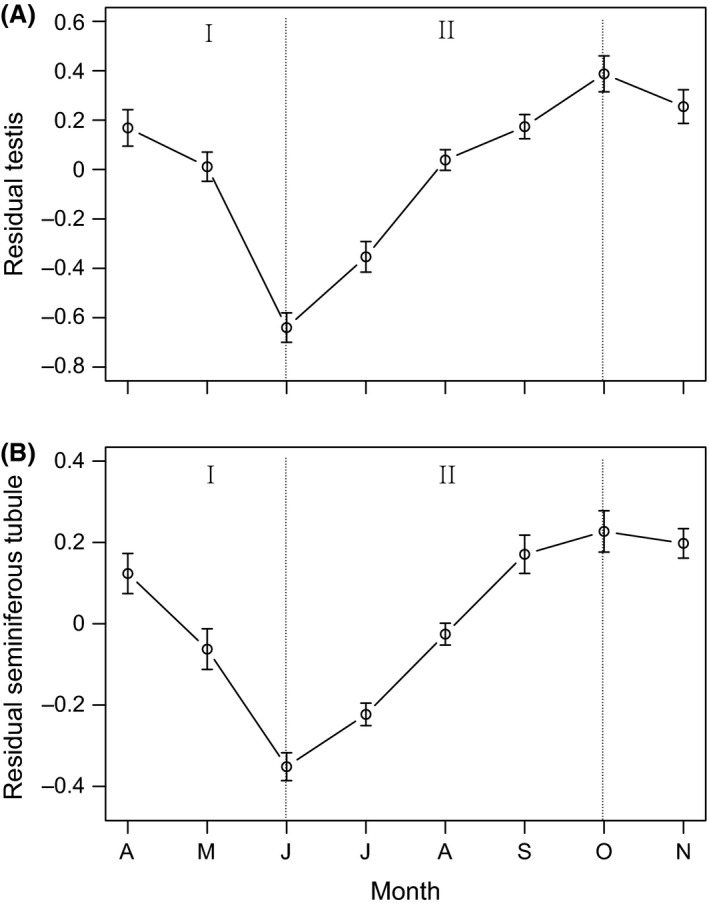
Monthly variation of (A) testis size, and (B) seminiferous tubule diameter in *P. vlangalii*. Testis size and seminiferous tubule diameter are quantified by the residuals from regressions of log_10_ (testis size) and log_10_ (seminiferous tubule diameter) against log_10_ (SVL). (I) Spermiation period; (II) Spermatogenesis period.

**Figure 4:**
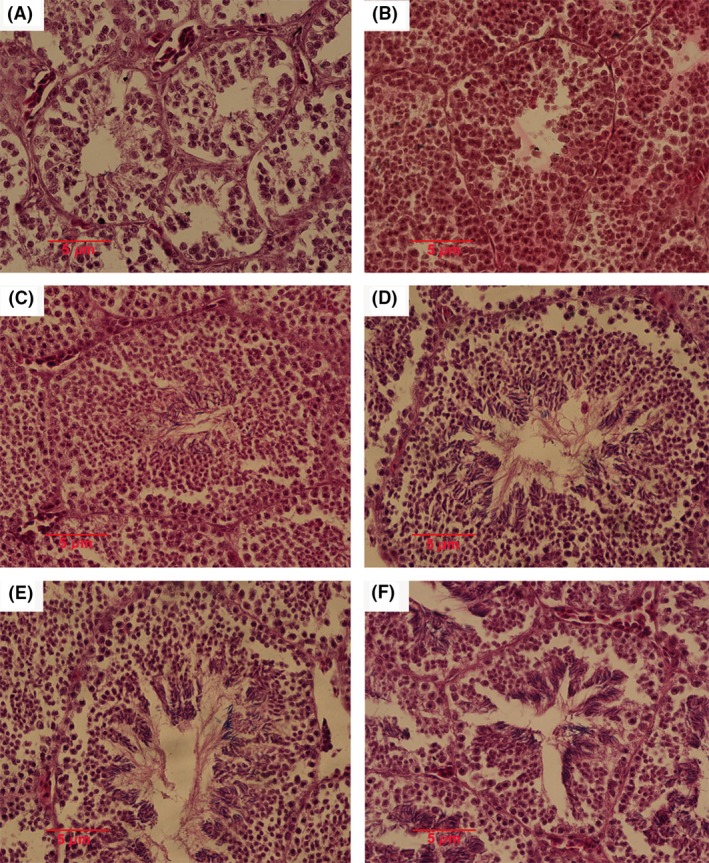
Male spermatogenesis stage of *P. vlangalii*. (A) Spermatogonia appeared in June (Stage I); (B) Spermatocytes appeared in August (Stage II); (C) Spermatids and sperm appeared in September (Stage III); (D)/(E) Sperms became abundant in October/November (Stage IV); (F) Abundant sperm before spermiation in May of the following year (Stage V).

For males, the testis size and diameter of seminiferous tubule decreased sharply from April to June, then increased gradually until September, and appeared to remain at maximum size during hibernation, because no significant change was observed after hibernation (Fig. 3). Testis size and diameter of seminiferous tubule varied significantly among months, while SVL was controlled for (testis size: month, *F*_7, 63_ = 35.51, *P *<* *0.001, SVL, *F*_1, 56_ = 132.99, *P *<* *0.001; seminiferous tubule diameter: month, *F*_7, 63_ = 48.01, *P *<* *0.001, SVL, *F*_1, 56_ = 5.39, *P *=* *0.02). The spermatogenesis stage was consistent with the variation of testis size and seminiferous tubule diameter (Fig. 4). In June, the seminiferous tubules consisted entirely of spermatogonia (Stage I), while both spermatogonia and spermatocytes could be found in August (Stage II), and spermatids became abundant in September (Stage III). The peak of spermiogenesis occurred in October, with a great number of spermatids and sperms observed in seminiferous tubules (Stage IV). During April and May of the next year, the spermatogenesis declined sharply (Stage V).

**Figure 5:**
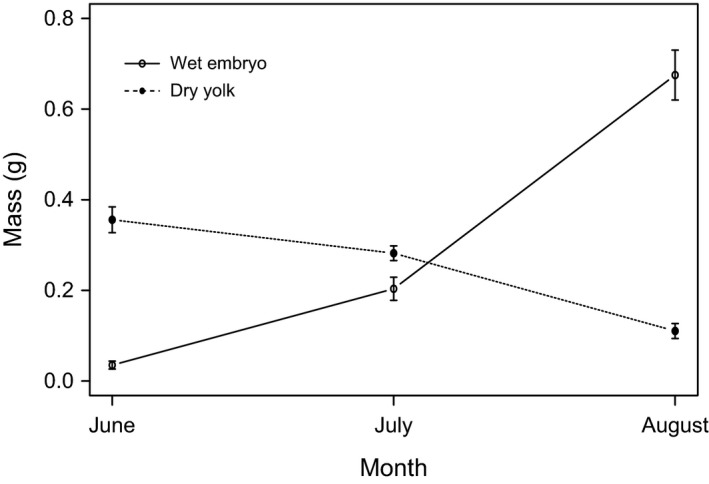
Monthly variation of female embryo wet mass and yolk dry mass in *P. vlangalii*.

**Figure 6:**
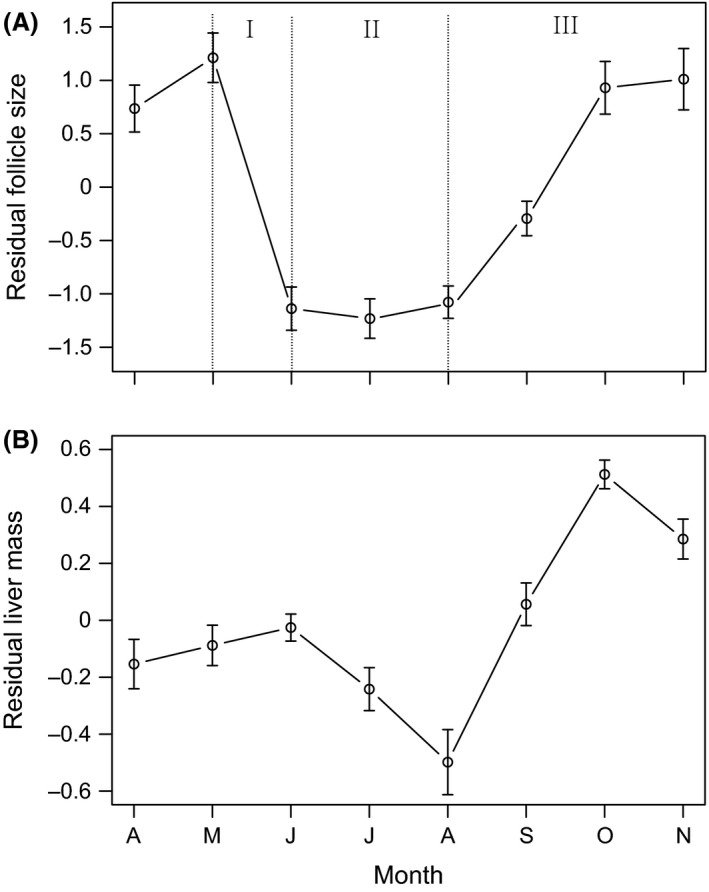
Monthly variation of (A) Female follicle size, and (B) Liver mass in *P. vlangalii*. Follicle size and liver mass are quantified as residuals from regressions of log_10_ (follicle size) and log_10_ (liver mass) against log_10_ (SVL). (I) Ovulation and fertilization period; (II) pregnancy period; (III) vitellogenesis period.

For females, enlarged follicles became embryos in late May and continually grew in mass from June to August, meanwhile the yolk mass decreased gradually from June to August, suggesting fertilization occurs in May (Fig. 5). Follicles were reduced in size through June–August. Vitellogenesis began in late August and continued through December. Follicle growth reached a maximum between September and October, and appeared to remain at maximum size from November to March. All adult females were synchronized in the same reproductive condition each month (Fig. 6A). At the same time, the liver mass decreased gradually from June to August, then increased sharply from August to October, and remained at high level during hibernation (Fig. 6B). The ANCOVA found significant monthly variation in follicle size and liver mass (follicle size: month, *F*_7, 62_ = 108.2, *P *<* *0.001, SVL, *F*_1, 55_ = 270.66, *P *<* *0.001; liver mass: month, *F*_7, 62_ = 53.76, *P *<* *0.001, SVL, *F*_1, 55_ = 149.27, *P *<* *0.001).

### Sexual maturity

The minimum SVL for adult male was 53 mm, while the minimum SVL for adult female was 51 mm. The average SVL for adult male was 58.04 ± 2.33 mm (*n* = 72), while the average of adult female was 56.56 ± 2.58 mm (*n* = 71). Adult males were significantly larger than adult females (*t *=* *6.23, *P *<* *0.001, df = 12.54). For subadults, the average SVL of males was 45.14 ± 1.81 mm (42.8–47.3 mm, *n *=* *10), and females exhibited an average of 41.67 ± 1.04 mm (40.23–43.12 mm, *n *=* *8) in SVL. For juveniles, the average SVL was 26.86 ± 1.17 mm (*n *=* *15). The adult male and female were significantly larger than subadult males and females (*P *<* *0.001), respectively. Meanwhile, the SVL of subadult male and female was significantly larger than these of neonates (*P *<* *0.001), suggesting that males and females required at least 2 years to reach sexual maturity. This was corroborated by mark–recapture study, that average SVL of neonates was 28.41 ± 0.26 mm (*n* = 16) in 2013, while in 2014, males exhibited an average of 42.79 ± 2.35 mm (*n* = 8) in SVL, and female showed an average of 40.71 ± 1.25 mm (*n* = 8). In 2015, the average SVL of males was 53.09 ± 1.48 mm (*n* = 8), while the average SVL of females was 51.11 ± 1.31 mm (*n* = 8), which have reached the size of sexual maturity.

### Male reproductive behaviors and offspring fitness

After emergence from hibernation in late April, males first established territory. Courtship and mating behavior occurred synchronously with female emergence in early May. This period lasted till early June. The first courtship case was observed on May 14th in 2012, and the last case was observed on June 10th. The frequency of courtship and mating behavior decreased gradually from May to June, and no reproductive behavior was observed after June. The parturition was synchronous and lasted from August 28th to September 12th. Hibernation began in late October, and no lizard was observed out of burrow after November 20th. We captured 96 neonates from 39 females in the enclosures, with mean litter size of 2.46 ± 0.88 (*n* = 39) ranging from 1 to 4. The average SVL of neonates was 26.12 ± 0.99 mm (*n* = 39), while the average mass of neonates mass was 0.90 ± 0.10 g (*n* = 39). Large females produced large neonates both in mass (*r *=* *0.38, *P *=* *0.016, df = 37) and SVL (*r *=* *0.42, *P *=* *0.007, df = 37), while no relationship was detected between female SVL and litter size (*r *=* *0.19, *P *=* *0.25, df = 37). The average RCM was 0.37 ± 0.14 (*n* = 39), with a range from 0.12 to 0.62. In April 2013, 81 neonates were re‐captured. The overall over‐winter survival rate of neonates was 84.4%. No significant and positive relationship was found between female SVL and offspring survival (*r *=* *0.21, *P *=* *0.19, df = 37).

## Discussion

Intersexual synchronous and annual reproduction clearly demonstrates that *P. vlangalii* exhibits an atypical reproductive cycle. Females start vitellogenesis in autumn, retain their follicles in utero during winter, and ovulate in spring of the next year. Parturition occurs in mid‐autumn, 2 months before hibernation. Male spermatogenesis occurs in summer and autumn, and spermiation happens simultaneously with ovulation in spring of the next year. All adult males and females are synchronized in the same reproductive condition each month. This reproductive cycle is atypical, compared to several montane viviparous lizards that show asynchronous reproductive cycles (e.g., *E. copei*, from Eje Neovolcanico, Mexico, Ramirez‐Bautista et al. 1996; *S. grammicus* from Hidalgo, Mexico, Hernández‐Salinas et al. 2010; *S. minor* from La Manzana of Hidalgo, Mexico, Ramirez‐Bautista et al. 2014), or exhibit synchronous but biannual or annual–biennial female reproductive cycles (*P. punae*, Boretto et al. 2007; *P. zapalensis*, Boretto and Ibarguengoytia 2009; *L. sarmientoi*, Fernandez et al. 2015).

The atypical reproductive cycle of *P. vlangalii* likely represents an alternative reproduction strategy among viviparous lizards living in cold environments of high altitudes (Roig et al. 2000; Medina and Ibargüengoytía 2010; Ramírez‐Bautista et al. 2012; Fernandez et al. 2015). Similar reproductive cycle has been observed in several viviparous lizards in highlands of Mexico and Patagonia, including *L. lineomaculatus* (Medina and Ibargüengoytía 2010), *S. grammicus* (Ramírez‐Bautista et al. 2012), and *L. sarmientoi* when females give birth earlier (Fernandez et al. 2015). Variations of reproductive cycle are generally associated with abiotic factors, such as precipitation, temperature, and photoperiod. While spermatogenesis is often positively associated with temperature, the vitellogenesis is more correlated with precipitation (Shine 2005; Ramírez‐Pinilla et al. 2009; Clusella‐Trullas et al. 2011). In temperate zone, most viviparous lizards exhibit an autumn reproductive cycle, in which females begin vitellogenesis in summer, ovulate in autumn, gestate in winter, give birth in spring and summer of the next year (Guillette and Casas‐Andreu 1980; Jiménez‐Cruz et al. 2005; Ramírez‐Pinilla et al. 2009). This reproductive cycle provides offspring abundant time for foraging and growth before winter (Ramirez‐Bautista et al. 1996; Jiménez‐Cruz et al. 2005). Meanwhile, females also gain ample food and store enough energy for follicle development and pregnancy in next cycle (Goldberg 1971; Guillette and Casas‐Andreu 1980; Ramirez‐Bautista et al. 1996). In cold climate of high altitudes, such as the Qinghai‐Tibetan Plateau, the winter season is long and temperature is often below −15°C (Fig. 2). The harsh climatic conditions likely influence the gestation and embryo viability (Atkins et al. 2007); therefore, females tend to accelerate gestation during favorable seasons without vitellogenesis and give birth in autumn before winter (Boretto et al. 2007; Boretto and Ibarguengoytia 2009). Meanwhile, neonates face a long time in hibernation almost immediately after birth without sufficient time to grow and acquire sufficient energy storage to survive (Medina and Ibargüengoytía 2010). Such harsh environment may promote the evolution of diverse strategies for offspring over‐winter survival (Ruby 1977; Shine 2006; Boretto et al. 2007). For example, newborns of *P. punae* exhibit large fat bodies and intra‐abdominal yolk (Boretto et al. 2007), and pregnant females of several viviparous lizards can adjust their thermal behaviors to maintain higher and stable body temperatures than their environments (Ruby 1977; Shine 2006).

The high over‐winter survival rate of neonates in *P. vlangalii* suggests that autumn parturition did not generate high costs to this atypical reproductive cycle. Compared to the over‐winter survival of other viviparous lizards (e.g., *N. ocellatus*, Uller et al. 2011, 41.5% for highland population; 30.7% for lowland populations), *P. vlangalii* had a higher over‐winter survival rate (84.4%). Several life history traits may have contributed to the neonates over‐winter survival. First, *P. vlangalii* has a delayed sex maturity age, and late sexual maturity allows female to reach sexual maturity at large body size and to give birth to large offspring (Piantoni et al. 2006; Boretto et al. 2015). *P. vlanglaii* needs at least 2 years to reach sexual maturity, while for other low‐elevation *Phrynocephalus* species, for example, *P. przewalskii* and *P. grumgrzimailoi*, offspring can reach sex maturity in 10–11 months (Liu et al. 2012; Zhao and Liu 2014). The minimum SVL for mature male and female *P. vlangalii* was 53 mm and 51 mm, respectively, much larger than these of other *Phrynocephalus* lizards from low‐elevation areas, for example, 45 mm and 43 mm, respectively, for male and female in *P. przewalskii* (Zhao and Liu 2014), and 48 mm and 47 mm, respectively, for male and female in *P. grumgrzimailoi* (Liu et al. 2012). Heavy and large offspring have higher fitness compared to small offspring, especially for species from high‐elevation areas (Piantoni et al. 2006; Jin and Liu 2007; Li et al. 2014). The positive (*r *=* *0.21) relationship between female body size and offspring over‐winter survival in *P. vlangalii* suggested the contribution of female body size to offspring survival, although statistically not significant. The lower significance might be constrained by our small sample size. Second, female *P. vlangalii* allocate a large amount of energy to embryonic development. This is evidenced by the female asynchronous gestation and vitellogenesis, and the large RCM. Embryonic development needs much energy (Ortiz et al. 2001), and metabolic cost of maintaining pregnancy is high (Robert and Thompson 2000). In addition, endocrinal factors may also constrain the simultaneous vitellogenesis and gestation (Custodia‐Lora and Callard 2002). Female *P. vlangalii* ceased vitellogenesis after ovulation and probably allocates much energy to embryo development as many viviparous lizards often do, such as *S. grammicus microlepidotus* (Guillette and Casas‐Andreu 1980), *S. mucronatus* (Estrada‐Flores et al. 1990), and *E. copei* (Ramirez‐Bautista et al. 1996). In addition, *P. vlangalii* exhibited a larger RCM of 0.37, compared to other viviparous lizards, for example, 0.23 for *Xenosaurus grandis rackhami* (Zamora‐Abrego et al. 2007) and 0.22 for *Eulamprus tympanum* (Doughty and Shine 1998), which corroborated that a large amount of energy was invested in reproduction by female *P. vlangalii*. Third and more importantly, neonates of *P. vlangalii* are known to share over‐winter burrows with adults (Qi et al. 2012), which may be the most important contributor to the high offspring over‐winter survival. A suitable burrow is essential for offspring survival in the long, cold, and snowy Zoige winter, especially for those neonates given birth at the end of the year (Medina and Ibargüengoytía 2010). Qi et al. (2012) found that adults of *P. vlangalii* shared burrows with nonrelated neonates, which is different from kin‐based associations in *Egernia‐Liopholis* clade of Australian skinks (Chapple 2003). This behavior likely protects offspring against predictors (Sinn et al. 2008), and more importantly, maintain high and stable environment temperature for offspring (Ruby 1977; Shine 2006). Similar burrow sharing behavior between adults and neonates has been observed in lizard *L. elongates* (Halloy et al. 2007).

Although females delayed their vitellogenesis during embryonic development, both spermatogenesis and vitellogenesis complete simultaneously in spring of the next year. Apparently, females are capable of accumulating sufficient energy for ovum recrudescing within a short time period. Our field observations suggested that two potential mechanisms may account for the female efficient energy accumulation. First, females may allocate more time in foraging after parturition compared to males. Second, females may postpone the time to hibernation, thereby prolong the foraging time. These hypotheses still need to be tested in future studies.

In summary, *P. vlangalii* exhibits an atypical reproduction cycle, which may represents an adaptation to its high‐elevation environments, because of its high offspring over‐winter survival. We argue that delayed sex maturity, asynchronous gestation and vitellogenesis, large RCM, and adult‐offspring burrow sharing behaviors have likely contributed to the success.

## Conflict of Interest

None declared.

## References

[ece31783-bib-0001] Agrawal, A. F. , E. D. Brodie , and J. Brown . 2001 Parent‐offspring coadaptation and the dual genetic control of maternal care. Science 292:1710–1712. 11387474 10.1126/science.1059910

[ece31783-bib-0002] Atkins, N. , R. Swain , E. Wapstra , and S. M. Jones . 2007 Late stage deferral of parturition in the viviparous lizard *Niveoscincus ocellatus* (Gray 1845): implications for offspring quality and survival. Biol. J. Linn. Soc. 90:735–746.

[ece31783-bib-0003] Boretto, J. M. , and N. R. Ibarguengoytia . 2006 Asynchronous spermatogenesis and biennial female cycle of the viviparous lizard *Phymaturus antofagastensis* (Liolaemidae): reproductive responses to high altitudes and temperate climate of Catamarca, Argentina. Amphib.‐Reptil. 27:25–36.

[ece31783-bib-0004] Boretto, J. M. , and N. R. Ibarguengoytia . 2009 *Phymaturus* of Patagonia, Argentina: reproductive biology of *Phymaturus zapalensis* (Liolaemidae) and a comparison of sexual dimorphism within the genus. J. Herpetol. 43:96–104.

[ece31783-bib-0005] Boretto, J. M. , N. R. Ibarguengoytia , J. C. Acosta , G. M. Blanco , J. Villavicencio , and J. A. Marinero . 2007 Reproductive biology and sexual dimorphism of a high‐altitude population of the viviparous lizard *Phymaturus punae* from the Andes in Argentina. Amphib.‐Reptil. 28:427–432.

[ece31783-bib-0006] Boretto, J. M. , F. Cabezas‐Cartes , and N. R. Ibargüengoytía . 2015 Energy allocation to growth and reproduction in a viviparous lizard endemic to the highlands of the Andes, Argentina. J. Zool. 297:77–86.

[ece31783-bib-0007] Chapple, D. G. 2003 Ecology, life‐history, and behavior in the Australian scincid genus *Egernia,* with comments on the evolution of complex sociality in lizards. Herpetol. Monogr. 17:145–180.

[ece31783-bib-0008] Clusella‐Trullas, S. , T. M. Blackburn , and S. L. Chown . 2011 Climatic predictors of temperature performance curve parameters in ectotherms imply complex responses to climate change. Am. Nat. 177:738–751. 21597251 10.1086/660021

[ece31783-bib-0009] Custodia‐Lora, N. , and I. P. Callard . 2002 Progesterone and progesterone receptors in reptiles. Gen. Comp. Endocrinol. 127:1–7. 12161195 10.1016/s0016-6480(02)00030-8

[ece31783-bib-0010] Doughty, P. , and R. Shine . 1998 Reproductive energy allocation and long‐term energy stores in a viviparous lizard (*Eulamprus tympanum*). Ecology 79:1073–1083.

[ece31783-bib-0011] Estrada‐Flores, E. , M. V.‐S. Cruz , F. R. Mendez‐de La Cruz , and G. Casas‐Andreu . 1990 Gonadal changes throughout the reproductive cycle of the viviparous lizard *Sceloporus mucronatus* (Sauria: Iguanidae). Herpetologica 46:43–50.

[ece31783-bib-0012] Farias, C. F. , R. A. Azevedo , and L. Brito‐Gitirana . 2006 Expression pattern of glycoconjugates in the Bidderian and ovarian follicles of the Brazilian toad *Bufo ictericus* analyzed by lectin histochemistry. Braz. J. Biol. 66:45–51. 16680305 10.1590/s1519-69842006000100007

[ece31783-bib-0013] Fernandez, J. B. , M. Medina , E. L. Kubisch , A. A. Manero , J. A. Scolaro , and N. R. Lbarguengoytia . 2015 Female reproductive biology of the lizards *Liolaemus sarmientoi* and *L. magellanicus* from the southern end of the world. Herpetol. J. 25:101–108.

[ece31783-bib-0014] Gavaud, J. 1986 Vitellogenesis in the lizard *Lacerta vivipara* Jacquin: II. Vitellogenin synthesis during the reproductive cycle and its control by ovarian steroids. Gen. Comp. Endocrinol. 63:11–23. 3770443 10.1016/0016-6480(86)90176-0

[ece31783-bib-0015] Goldberg, S. R. 1971 Reproductive cycle of the oviviparous iguanid lizard *Sceloporus jarrovi* Cope. Herpetologica 27:123–131.

[ece31783-bib-0016] Goldberg, S. R. , and C. H. Lowe . 1966 The reproductive cycle of the western whiptail lizard (*Cnemidophorus tigris*) in southern Arizona. J. Morphol. 118:543–548. 5956247 10.1002/jmor.1051180407

[ece31783-bib-0017] Guillette, J. L. J. , and G. Casas‐Andreu . 1980 Fall reproductive activity in the high altitude Mexican lizard, *Sceloporus grammicus microlepidotus* . J. Herpetol. 14:143–147.

[ece31783-bib-0018] Halloy, M. , J. M. Boretto , and N. R. Ibargüengoytía . 2007 Signs of parental behavior in *Liolaemus elongatus* (Sauria: Liolaemidae) of Neuquen, Argentina. South. Am. J. Herpetol. 2:141–147.

[ece31783-bib-0019] Hernández‐Gallegos, O. , F. R. Mendez‐De La Cruz , M. Villagrán‐Santa Cruz , and R. M. Andrews . 2002 Continuous spermatogenesis in the lizard *Sceloporus bicanthalis* (Sauria: Phrynosomatidae) from high elevation habitat of central Mexico. Herpetologica 58:415–421.

[ece31783-bib-0020] Hernández‐Salinas, U. , A. Ramírez‐Bautista , A. Leyte‐Manrique , and G. R. Smith . 2010 Reproduction and sexual dimorphism in two populations of *Sceloporus grammicus* (Sauria: Phrynosomatidae) from Hidalgo, Mexico. Herpetologica 66:12–22.

[ece31783-bib-0021] Ibargüengoytía, N. R. 2004 Prolonged cycles as a common reproductive pattern in viviparous lizards from Patagonia, Argentina: reproductive cycle of *Phymaturus patagonicus* . J. Herpetol. 38:73–79.

[ece31783-bib-0022] Jiménez‐Cruz, E. , A. Ramírez‐Bautista , J. C. Marshall , M. Lizana‐Avia , A. Nieto‐Montes De Oca , and G. C. Carpenter . 2005 Reproductive cycle of *Sceloporus grammicus* (Squamata: Phrynosomatidae) from Teotihuacan, Mexico. Southwest. Nat. 50:178–187.

[ece31783-bib-0023] Jin, Y. T. , and N. F. Liu . 2007 Altitudinal variation in reproductive strategy of the toad‐headed lizard, *Phrynocephalus vlangalii* in North Tibet Plateau (Qinghai). Amphib.‐Reptil. 28:509–515.

[ece31783-bib-0024] Jones, S. M. , E. Wapstra , and R. Swain . 1997 Asynchronous male and female gonadal cycles and plasma steroid concentrations in a viviparous lizard, *Niveoscincus ocellatus* (Scincidae), from Tasmania. Gen. Comp. Endocrinol. 108:271–281. 9356222 10.1006/gcen.1997.6971

[ece31783-bib-0025] Li, J. Q. , R. Zhou , and N. F. Liu . 2014 Life‐history variation among three populations of the toad‐headed lizard *Phrynocephalus vlangalii* along an elevation gradient on the northeastern Tibetan Plateau. Herpetol. J. 24:17–23.

[ece31783-bib-0026] Liu, Y. , Y. Song , W. Li , and L. Shi . 2012 Reproductive strategy and cycle of the toad‐headed agama *Phrynocephalus grumgrzimailoi* (Agamidae) in Xinjiang, China. Asian Herpetol. Res. 3:198–204.

[ece31783-bib-0027] Martin, P. , and P. Bateson . 2007 Measuring behaviour: an introductory guide. Cambridge Univ. Press, Cambridge.

[ece31783-bib-0028] Medina, M. , and N. R. Ibargüengoytía . 2010 How do viviparous and oviparous lizards reproduce in Patagonia? A comparative study of three species of *Liolaemus* . J. Arid Environ. 74:1024–1032.

[ece31783-bib-0029] Olsson, M. , and R. Shine . 1999 Plasticity in frequency of reproduction in an alpine lizard, *Niveoscincus microlepidotus* . Copeia 3:794–796.

[ece31783-bib-0030] Ortiz, M. F. , A. N.‐M. de Oca , and I. H. S. Ugarte . 2001 Diet and reproductive biology of the viviparous lizard *Sceloporus torquatus torquatus* (Squamata: Phrynosomatidae). J. Herpetol. 35:104–112.

[ece31783-bib-0031] Piantoni, C. , N. R. Ibarguengoytia , and V. E. Cussac . 2006 Growth and age of the southernmost distributed gecko of the world (*Homonota darwini*) studied by skeletochronology. Amphib.‐Reptil. 27:393–400.

[ece31783-bib-0032] Pilorge, T. 1987 Density, size structure, and reproductive characteristics of three populations of *Lacerta vivipara* (Sauria: Lacertidae). Herpetologica 43:345–356.

[ece31783-bib-0033] Powell, G. L. , and A. P. Russell . 1991 Parturition and clutch characteristics of short‐horned lizards (*Phrynosoma douglassii brevirostre*) from Alberta. Can. J. Zool. 69:2759–2764.

[ece31783-bib-0034] Qi, Y. , D. W. Noble , J. Fu , and M. J. Whiting . 2012 Spatial and social organization in a burrow‐dwelling lizard (*Phrynocephalus vlangalii*) from China. PLoS One 7:e41130. 22844434 10.1371/journal.pone.0041130 PMC3402523

[ece31783-bib-0035] R Development Core Team . 2011 R: A language and environment for statistical computing. R Foundation for Statistical Computing, Vienna, Austria.

[ece31783-bib-0036] Radder, R. , B. Shanbhag , and S. Saidapur . 2001 Pattern of plasma sex steroid hormonal levels during reproductive cycles of male and female tropical lizard, *Calotes versicolor* . Gen. Comp. Endocrinol. 124:285–292. 11742511 10.1006/gcen.2001.7711

[ece31783-bib-0037] Ramírez‐Bautista, A. , and V. Olvera‐Becerril . 2004 Reproduction in the boulder spiny lizard, *Sceloporus pyrocephalus* (Sauria: Phrynosomatidae), from a tropical dry forest of Mexico. J. Herpetol. 38:225–231.

[ece31783-bib-0038] Ramírez‐Bautista, A. , and L. J. Vitt . 1997 Reproduction in the lizard *Anolis nebulosus* (Polychrotidae) from the Pacific coast of Mexico. Herpetologica 53:423–431.

[ece31783-bib-0039] Ramirez‐Bautista, A. , L. J. Jr Guillette , G. Gutierrez‐Mayen , and Z. Uribe‐Peña . 1996 Reproductive biology of the lizard *Eumeces copei* (Lacertilia: Scincidae) from the Eje Neovolcanico, Mexico. Southwest. Nat. 41:103–110.

[ece31783-bib-0040] Ramirez‐Bautista, A. , J. Barba‐Torres , and L. J. Vitt . 1998 Reproductive cycle and brood size of *Eumeces lynxe* from Pinal de Amoles, Queretero, México. J. Herpetol. 32:18–24.

[ece31783-bib-0041] Ramirez‐Bautista, A. , L. J. Vitt , A. Ramirez‐Hernandez , F. M. Quijano , and G. R. Smith . 2008 Reproduction and sexual dimorphism of *Lepidophyma sylvaticum* (Squamata: Xantusiidae), a tropical night lizard from Tlanchinol, Hidalgo, Mexico. Amphib.‐Reptil. 29:207–216.

[ece31783-bib-0042] Ramirez‐Bautista, A. , B. P. Stephenson , C. S. Munoz , R. Cruz‐Elizalde , and U. Hernandez‐Salinas . 2014 Reproduction and sexual dimorphism in two populations of the polymorphic spiny lizard *Sceloporus minor* from Hidalgo, Mexico. Acta Zool. 95:397–408.

[ece31783-bib-0043] Ramírez‐Bautista, A. , R. García‐Collazo , L. J. Jr Guillette , and G. C. Carpenter . 2006 Reproductive, fat, and liver cycles of male and female rose‐bellied lizards, *Sceloporus variabilis*, from coastal areas of southern Veracruz, Mexico. Southwest. Nat. 51:163–171.

[ece31783-bib-0044] Ramírez‐Bautista, A. , B. P. Stephenson , A. Lozano , H. Uribe‐Rodríguez , and A. Leyte Manrique . 2012 Atypical reproductive cycles in a population of *Sceloporus grammicus* (Squamata: Phrynosomatidae) from the Mexican Plateau. Ecol. Evol. 2:1903–1913. 22957191 10.1002/ece3.310 PMC3433993

[ece31783-bib-0045] Ramírez‐Bautista, A. , B. P. Stephenson , C. Serrano Muñoz , R. Cruz‐Elizalde , and U. Hernández‐Salinas . 2013 Reproduction and sexual dimorphism in two populations of the polymorphic spiny lizard *Sceloporus minor* from Hidalgo, México. Acta Zool. 95:397–408.

[ece31783-bib-0046] Ramírez‐Pinilla, M. P. , M. L. Calderón‐Espinosa , O. Flores‐Villela , A. Muñoz‐Alonso , and F. R. Méndez de la Cruz . 2009 Reproductive activity of three sympatric viviparous lizards at Omiltemi, Guerrero, Sierra Madre del Sur, Mexico. J. Herpetol. 43:409–420.

[ece31783-bib-0047] Robert, K. A. , and M. B. Thompson . 2000 Energy consumption by embryos of a viviparous lizard, *Eulamprus tympanum*, during development. Comp. Biochem. Physiol. A 127:481–486. 10.1016/s1095-6433(00)00278-6 11154944

[ece31783-bib-0048] Roff, D. A. 2002 Life history evolution. Sinauer Associates Inc, Sunderland.

[ece31783-bib-0049] Roig, J. M. , M. A. Carretero , and G. A. Llorente . 2000 Reproductive cycle in a pyrenean oviparous population of the common lizard (*Zootoca vivipara*). Neth. J. Zool. 50:15–27.

[ece31783-bib-0050] Ruby, D. E. 1977 Winter activity in yarrow's spiny lizard, *Sceloporus jarrovi* . Herpetologica 33:322–333.

[ece31783-bib-0051] Selby, S. M. 1965 Standard mathematical tables. Chemical Rubber Co, Cleveland.

[ece31783-bib-0052] Shine, R. 1992 Relative clutch mass and body shape in lizards and snakes: is reproductive investment constrained or optimized? Evolution 46:828–833. 10.1111/j.1558-5646.1992.tb02088.x 28568652

[ece31783-bib-0053] Shine, R. 2005 Life‐history evolution in reptiles. Annu. Rev. Ecol. Evol. Syst. 36:23–46.

[ece31783-bib-0054] Shine, R. 2006 Is increased maternal basking an adaptation or a pre‐adaptation to viviparity in lizards? J. Exp. Zool. 305A:524–535. 10.1002/jez.a.291 16555302

[ece31783-bib-0055] Sinn, D. L. , G. M. While , and E. Wapstra . 2008 Maternal care in a social lizard: links between female aggression and offspring fitness. Anim. Behav. 76:1249–1257.

[ece31783-bib-0056] Uller, T. , G. M. While , C. D. Cadby , A. Harts , K. O'Connor , I. Pen , et al. 2011 Altitudinal divergence in maternal thermoregulatory behaviour may be driven by differences in selection on offspring survival in a viviparous lizard. Evolution 65:2313–2324. 21790577 10.1111/j.1558-5646.2011.01303.x

[ece31783-bib-0057] Vial, J. L. , and J. R. Stewart . 1985 The reproductive cycle of *Barisia monticola*: a unique variation among viviparous lizards. Herpetologica 41:51–57.

[ece31783-bib-0058] Wu, P. F. , Y. Z. Wang , S. G. Wang , T. Zeng , H. X. Cai , H. Y. Guo , et al. 2002 The age structure and sex ratio of *Phrynocephalus vlangalii* (Sauria: Agamidae). J. Sichuan Univ. 39:1134–1139.

[ece31783-bib-0059] Zamora‐Abrego, J. G. , J. J. Zuñiga‐Vega , and A. Nieto‐Montes de Oca . 2007 Variation in reproductive traits within the lizards genus *Xenosaurus* . J. Herpetol. 41:630–637.

[ece31783-bib-0060] Zhao, K. T. 1999 *Phrynocephalus* kaup Pp. 153–193 *in* Zhao E. M., Zhao K. T. and Zhou K. Y., eds. Fauna sinica, reptilia. Science Press, Beijing.

[ece31783-bib-0061] Zhao, W. , and N. F. Liu . 2014 The proximate causes of sexual size dimorphism in *Phrynocephalus przewalskii* . PLoS One 9:e85963. 24465815 10.1371/journal.pone.0085963 PMC3897606

